# High Molecular Weight Hyaluronic Acid Reduces the Expression of Virulence Genes fimA, mfa1, hagA, rgpA, and kgp in the Oral Pathogen *Porphyromonas gingivalis*

**DOI:** 10.3390/pharmaceutics14081628

**Published:** 2022-08-04

**Authors:** Meshal S. Alharbi, Fahad A. Alshehri

**Affiliations:** 1 Department of Periodontics and Community Dentistry, College of Dentistry, King Saud University, Riyadh 12372, Saudi Arabia; 2 Qassim Health Cluster, Ministry of Health, Buraydah 52367, Saudi Arabia

**Keywords:** periodontal disease, periodontitis, peri-mucositis, peri-implantitis, porphyromonas gingivalis, hyaluronic acid, sodium hyaluronate, azithromycin, chlorhexidine, gingipains

## Abstract

*Porphyromonas gingivalis* (*P. gingivalis*) is a cornerstone pathogen in the development and progression of periodontal and peri-implant tissue destruction. It is capable of causing dysbiosis of the microbial biofilm and modulation of the host immune system. Hyaluronic acid (HA) is a naturally occurring glycosaminoglycan found in all living organisms. It is well known and has been used for improving tissue healing. In addition, some studies have suggested that there may be an antimicrobial potential to HA. The aim of this study was to evaluate the effect of hyaluronic acid, azithromycin (AZM), and chlorhexidine (CHX) on the expression of genes (i.e., fimA, mfa1, hagA, rgpA, rgpB, and kgp) related to the virulence and adhesion of *P. gingivalis*. The study groups were divided into four: (1) HA treated group; (2) AZM treated group; (3) CHX treated group; and (4) untreated group to serve as a negative control. *P. gingivalis* ATCC 33277 was cultured and then exposed to four different concentrations (100% MIC, 50% MIC, 25% MIC, and 12.5% MIC) of HA, AZM, and CHX for 24 h. The expression levels of the aforementioned genes were measured using quantitative reverse transcription polymerase chain reaction (RT-qPCR). Relative fold-change values were calculated and compared between groups. The fold-change values of all genes combined were 0.46 ± 0.33, 0.31 ± 0.24, and 0.84 ± 0.77 for HA, AZM, and CHX, respectively. HA has downregulated all the genes by mostly a half-fold: 0.35 ± 0.20, 0.47 ± 0.35, 0.44 ± 0.25, 0.67 ± 0.46, 0.48 ± 0.33 and 0.35 ± 0.22 with fimA, mfa1, hagA, rgpA, rgpB and kgp, respectively. The effect of HA was significant on all genes except rgpB compared to the untreated control. Lower concentrations of HA tended to exhibit greater downregulation with 1 mg/mL being the most effective. High molecular weight (1.5 MDa) hyaluronic acid has a potent effect on *P. gingivalis* by downregulating fimA, mfa1, hagA, rgpA, and kgp. The effect of HA was generally less than that of AZM but greater than that of CHX.

## 1. Introduction

The role of microbial biofilm is well-established in the initiation and progression of periodontal and peri-implant diseases [1,2]. Our understanding of the etiology of periodontal diseases has broadened over the years [3]. It is now largely accepted that a complex interaction between microbial, genetic, environmental, nutritional, and other modifying factors is responsible for the shift in periodontal tissues from health to destruction [4]. Nevertheless, microbial products remain believed to be the trigger of immunoinflammatory mechanisms in this complex process [4]. Moreover, periodontal tissue destruction is usually associated with dominance of certain periodontal pathogens [5]. In line with this recent explanation, the theory of “keystone pathogen” implies that alongside the multitude of interacting factors, certain critical pathogens can alter the microbiota without requiring quantitative dominance [6]. Based on this concept, *Porphyromonas gingivalis* (*P. gingivalis*) has been shown to modify the biofilm and disrupt microbe-host homeostasis leading to the development of periodontitis [7]. It is also speculated that a keystone pathogen can directly remodel other species in the biofilm independently of host modulation [8].

*P. gingivalis* is a Gram-negative, black-pigmented, strictly anaerobic rod. It is largely regarded as a crucial pathogen in the etiology of periodontal diseases [9]. Among the different virulence factors exhibited by *P. gingivalis* are capsules of different serotypes [10], which allow for adhesion, resistance to phagocytosis, and decreased autoagglutination and stimulation of polymorphonuclear leukocyte chemiluminescence [9]. Fimbriae are proteinaceous appendages that protrude from the cell wall of *P. gingivalis*. These are key virulent structures that aid in adhesion to host cells and tissues. Fimbriae are expressed in at least two forms: long or major fimbria encoded by fimA and short or minor fimbria encoded by mfa1 [11]. Other virulence factors include the expression of lipopolysaccharides, proteases, and other outer membrane proteins [9]. Gingipains are proteolytic enzymes responsible for the degradation of host proteins and processing of fimbriae subunits [12]. Gingipains are classified into two types: (1) arg-gingipain which cleaves to arginine and is encoded by rgpA and rgpB, and (2) lys-gingipain which cleaves to lysine and is encoded by kgp. Hemagglutinin A gene (hagA) is involved in hemagglutinin activity and the promotion of co-aggregation with other bacteria [13].

While mechanical root surface debridement is typically sufficient for stabilizing periodontal tissues, further chemotherapy has always been required to improve treatment outcomes in some difficult cases and non-responding periodontal sites [14]. Current clinical practice guidelines acknowledge the additional benefits of using systemic or local antibiotics as adjunctive therapy for the treatment of periodontitis stages I-III [15]. While local antibiotics may be used, systemic antibiotics are not recommended for routine use due to concerns about their impact on patient and public health. Systemic antibiotics may, however, still be prescribed for special patient categories [15]. The administration of 2 g or 3 g of amoxicillin preoperatively was also shown to be beneficial for the prevention of implant failures [16]. The use of antibiotics is especially important in the case of peri-implant diseases as rough implant surfaces hinder mechanical biofilm removal [17,18]. With the rising concern of antibiotic resistance, the introduction of non-antibiotic substances having antimicrobial properties into periodontal/implant treatments warrants attention. Hyaluronic acid has been shown to carry such potential [19].

Hyaluronic acid (HA) or hyaluronan [20] is a non-protein, non-sulfated glycosaminoglycan constructed of glucuronic acid and N-acetylglucosamine disaccharide units [21]. HA contributes to the physical properties of tissues such as viscoelasticity. It has high moisture retention capacity, biocompatibility, and hygroscopic properties [22]. HA exerts an anti-inflammatory effect by suppressing the production of matrix metalloproteinases and the activity of interleukin-1β [23,24,25]. It can also induce an analgesic effect as it interferes with HA-receptors at free nerve endings [26,27]. HA has demonstrated osteoconductive capabilities by accelerating new bone formation in rats [28]. In addition, HA might have an antimicrobial effect by impairing both the growth and attachment of certain microbial species including *Staphylococcus aureus*, *Streptococcus mutans*, *Escherichia coli*, and *Pseudomonas aeruginosa* [19,21].

While a good number of studies have investigated HA properties in reducing inflammation and accelerating healing [29,30,31,32,33], only a few studies have looked into its antibacterial potential, especially against *P. gingivalis*. Back in 1999, Pirnazar et al. observed that HA has generally exerted a bacteriostatic effect on various periodontal pathogens. They described the effect of HA on *P. gingivalis* to be further enhanced by high molecular weight and greater concentrations [34]. A very recent in vitro study has observed that 0.8% HA was able to suppress *P. gingivalis* more effectively compared to 0.2% Chlorohexidine [35]. We take the research further by exploring the mechanism by which HA could suppress *P. gingivalis*. We investigate the effect of high molecular weight HA of multiple concentrations on the expression levels of virulent genes: fimA, mfa1, hagA, rgpA, rgpB, and kgp. We are also comparing HA to azithromycin and chlorhexidine.

Thus, the aim of this study is to evaluate the effect of hyaluronic acid, azithromycin, and chlorhexidine on the expression of genes (fimA, mfa1, hagA, rgpA, rgpB, and kgp) related to the virulence and adhesion of *P. gingivalis*.

## 2. Materials and Methods

The study protocol was approved by the College of Dentistry Research Center (CDRC) at King Saud University (CDRC registration number: PR0125). Laboratory procedures were carried out at the Molecular and Cell Biology Laboratory, College of Dentistry, King Saud University.

### 2.1. Culture Methods

*P. gingivalis* derived from ATCC 33277 (Microbiologics, Kwik-stik, 0912, Saint Cloud, MN, USA) was activated on a Brucella blood agar supplemented with hemin and vitamin K (Watin Biolife, 2046, Riyadh, Saudi Arabia). The agar plates were placed in an anaerobic jar (Thermo Scientific, Oxoid Anaerojar, AG0025, Basingstoke, Hants, UK) alongside a gas-generating sachet (Thermo Scientific, Oxoid AnaeroGen 2.5 L, AN0025A, Basingstoke, Hants, UK). The jar was incubated at 37 °C for 5 days. For later experiments, a modified tryptic soy broth (mTSB) was prepared by adding 30 mg/mL tryptic soy, 5 mg/mL yeast extract, 0.5 mg/mL L-cysteine hydrochloride, 5 µg/mL hemin, and 1 µg/mL menadione. All liquid media incubations were performed in a shaker at 140 RPM and 37 °C.

### 2.2. Minimum Inhibitory Concentration

To determine the minimum inhibitory concentration (MIC), mTSB was inoculated with *P. gingivalis* and adjusted to OD_600_ 0.08 on a spectrophotometer. Then, 100 µL of the suspension was distributed in a 96-well microplate (Thermo Scientific, Nunc MicroWell Plates, 163320, Roskilde, Zealand, Denmark). Following this, 8 mg/mL 1.5 MDa sodium hyaluronate (Lifecore Biomedical, HA15 M-5, Chaska, MN, USA) were two-fold serially diluted and incubated with the suspension. Additionally, 50 µg/mL azithromycin dihydrate (AK Scientific, G333, Union City, CA, USA) and 2 mg/mL Chlorhexidine Gluconate (Avalon Pharma, PS-2035, Riyadh, Saudi Arabia) were also two-fold serially diluted and included to serve as positive controls. Sodium chloride (9%) was used as a solvent for all three substances. The microwell plate was incubated as previously described for 24 h. Following incubation, 5 µL drops of each well were placed onto Brucella agar plates supplemented with hemin and vitamin K. The plates were incubated for 5 days to assess the growth visually from each concentration. The lowest concentration that had no or only minuscule growth was considered MIC. The procedures were conducted in triplicates.

### 2.3. Quantitative Reverse Transcription Polymerase Chain Reaction (RT-qPCR)

In a 24-well plate (Greiner, CELLSTAR^®^ Multiwell plate, 662160, Frickenhausen, Esslingen, Germany), 1 mL of mTSB suspension with *P. gingivalis* was placed in 4 wells. The *P. gingivalis* inoculum had been adjusted to an OD600 0.05 on a spectrophotometer. Each well containing the suspension was incubated with 100% MIC, 50% MIC, 25% MIC, and 12.5% MIC of HA. The same was repeated for AZM and CHX. Suspension without any antimicrobial was used as a control. The plate was incubated as previously described for 24 h. Following incubation, total RNA extraction was carried out following manufacturer instructions (BioFACT, Total RNA Prep Kit Ver.2.0, RP101-100, Yuseong-gu, Daejeon, Korea). Components of a complementary DNA synthesis kit (Solis BioDyne, FIREScript RT cDNA synthesis KIT, 06-15-00050, Tartu, Estonia) were mixed and added to the total RNA samples. Reverse transcription was conducted by performing the following protocol: primer annealing at 25 °C for 10 min, reverse transcription at 50 °C for 60 min, and enzyme inactivation at 85 °C for 5 min (Applied Biosystems, GeneAmp PCR System 9700, SG). For the qPCR step, primers were designed using the Primer-BLAST tool (National Center for Biotechnology Information, Bethesda, MD, USA) based on the reference sequence by Naito et al. [36] (Table 1). Primers (Macrogen, Geumcheon-gu, Seoul, Korea) were added to the master mix solution (Solis BioDyne, HOT FIREPol EvaGreen qPCR Supermix, 08-36-00001-5, Tartu, Estonia) and mixed with the cDNA samples in a microplate (Applied Biosystems, MicroAmp Optical 96-Well Reaction Plate, N8010560, CN) for a total 20 µL in each well. Amplification was carried out by initial activation for 12 min at 95 °C. Followed by 40 cycles of denaturation at 95 °C for 15 s, annealing at 60 °C for 30 s, and elongation at 72 °C for 30 s (Applied Biosystems, 7500 Real-Time PCR System, SG).

### 2.4. Statistical Analysis

The sample size was calculated using G*Power (version 3.1.9.6, Franz Faul, Kiel University, Kiel, Schleswig-Holstein, Germany). A sample size of 78 (6 in each group) achieves 87% power to detect a difference of 0.55 at a 0.05 significance level.

Cycle threshold (CT) values for 6 replicates of each sample were entered in a spreadsheet using Microsoft Excel for Microsoft 365 MSO (version 2205, Microsoft, Redmond, WA, USA). Fold change relative to control was calculated using the equation 2^−(ΔΔCT)^ in the spreadsheet.

Statistical analysis was carried out using IBM SPSS Statistics (version 20, IBM, Armonk, NY, USA). Means were reported as descriptive values. Welch and Brown-Forsythe corrected one-way analysis of variance were used to compare fold change values between different concentrations and different antimicrobials. Post HOC Games-Howell method was used to compare group means. A *p*-value of less than 0.05 was considered statistically significant. Data graphs were produced using Prism 9 (version 9.3.1, GraphPad Software, San Diego, CA, USA).

## 3. Results

### 3.1. Minimum Inhibitory Concentration (MIC)

**Figure 1:**
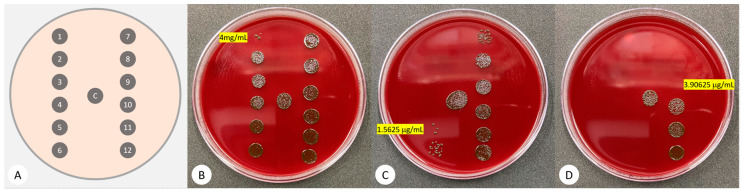
**(A)** A diagram depicting the distribution of 5 µL drops from 12 wells that were diluted by a two-fold factor. ‘C’ refers to untreated control; (**B**) Hyaluronic acid from 4 mg/mL to 2 µg/mL; (**C**) Azithromycin from 25 µg/mL to 0.012 µg/mL; (**D**) Chlorhexidine from 1 mg/mL to 0.49 µg/mL.

The observed MIC against *P. gingivalis* was 4 mg/mL for HA, 1.6 µg/mL for azithromycin, and 3.9 µg/mL for chlorhexidine. The working concentrations for this study were 100% MIC, 50% MIC, 25% MIC, and 12.5% MIC for each material which was reached by two-fold dilution (Figure 1).

### 3.2. Gene Expression

**Figure 2:**
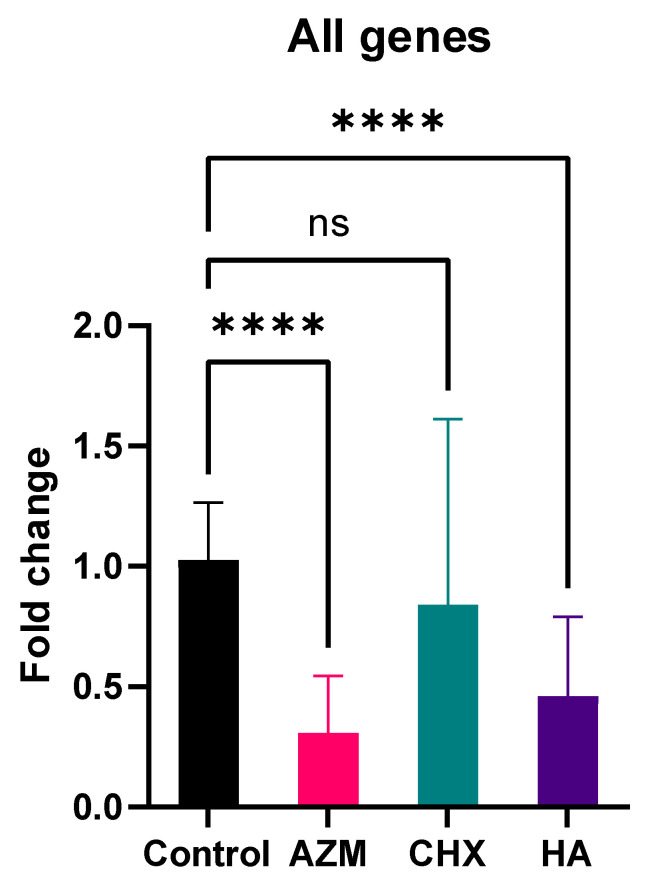
Overall fold-change values (mean ± SD) for all genes combined. >0.05 (ns), ≤0.05 (*), ≤0.01 (**), ≤0.001 (***), ≤0.0001 (****).

All three materials demonstrated downregulation of all genes combined regardless of concentration. CHX resulted in 0.84 ± 0.77 folds while both AZM and HA were less than half a fold at 0.31 ± 0.24 and 0.46 ± 0.33, consecutively. Only AZM and HA were statistically significant compared to the control (untreated group). Additionally, AZM mean fold-change value was significantly lower than both CHX and HA. HA was significantly lower than CHX (Table 2 and Figure 2).

#### 3.2.1. fimA

**Figure 3:**
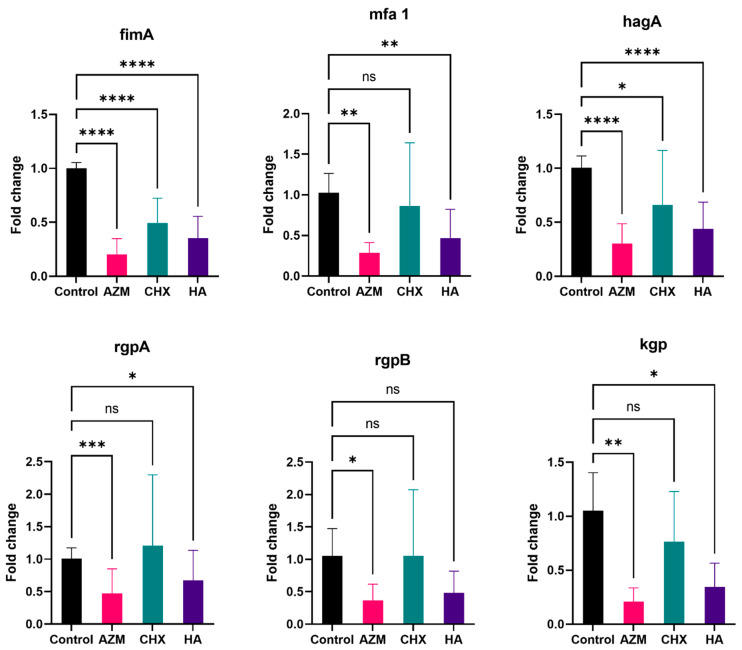
Overall fold-change values (mean ± SD) for independent genes. >0.05 (ns), ≤0.05 (*), ≤0.01 (**), ≤0.001 (***), ≤0.0001 (****).

Regardless of concentration, all materials showed significant downregulation of fimA. Induction of the gene was 0.20 ± 0.15, 0.49 ± 0.23, and 0.35 ± 0.20 the level of the control for AZM, CHX, and HA, consecutively. AZM demonstrated the greatest downregulation and was significantly lower than both CHX and HA. However, HA was not statistically significant compared to CHX (Table S1 and Figure 3).

**Figure 4:**
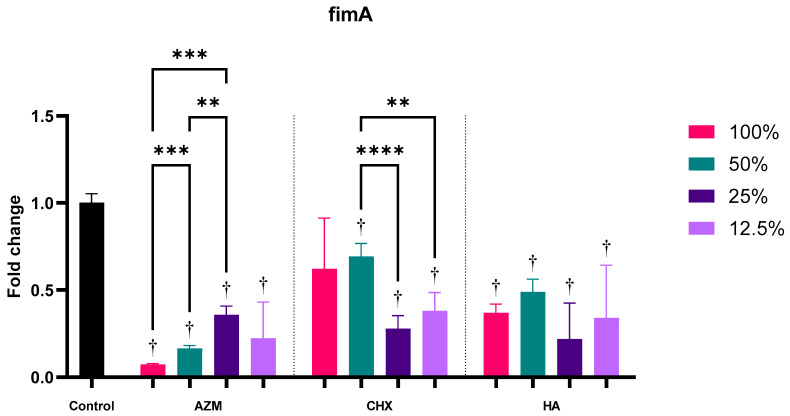
Fold-change values (mean ± SD) of different concentrations on expression of fimA. >0.05 (ns), ≤0.05 (*), ≤0.01 (**), ≤0.001 (***), ≤0.0001 (****). Statistically significant compared to control *p* < 0.05 (†).

All four concentrations of AZM have significantly reduced fimA expression. Higher concentrations of AZM showed a greater reduction. In fact, the higher two concentrations of AZM (100% and 50%) showed the greatest reduction among all materials at 0.07 ± 0.01 and 0.16 ± 0.02 folds. MIC concentration of CHX has reduced fimA expression but not significantly *p* = 0.285, while all sub-MIC concentrations of CHX showed significant reduction. All concentrations of HA reduced fimA expression significantly. Higher concentrations of HA had slightly less reduction than lower concentrations, but there was no significant difference between any of the concentrations of HA (Table S2 and Figure 4).

#### 3.2.2. mfa1

Generally, all three materials reduced the expression of mfa1 by 0.28 ± 0.13, 0.87 ± 0.78, and 0.47 ± 0.35 folds for AZM, CHX, and HA, consecutively. AZM and HA were significantly lower than the control. Additionally, AZM was significantly lower than CHX but not HA. CHX and HA were also not statistically significant compared to each other (Table S1 and Figure 3).

**Figure 5:**
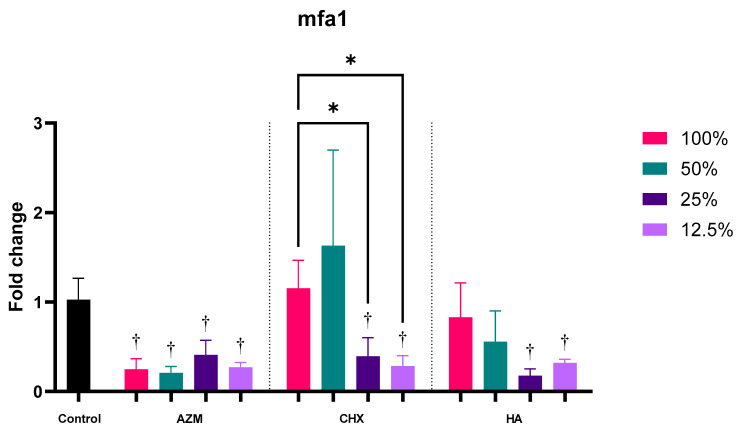
Fold-change values (mean ± SD) of different concentrations on expression of mfa1. >0.05 (ns), ≤0.05 (*), ≤0.01 (**), ≤0.001 (***), ≤0.0001 (****). Statistically significant compared to control *p* < 0.05 (†).

All concentrations of AZM significantly downregulated mfa1 expression with no significant difference between them. CHX, on the other hand, was able to reduce the expression of mfa1 with lower concentrations only; 25% and 12.5% by 0.39 ± 0.21 and 0.29 ± 0.12 folds, consecutively. Higher concentrations of CHX have actually increased the induction of mfa1, but not to a significant level. HA has reduced mfa1 gene expression at all concentrations, but only the lower two concentrations; 25% and 12.5% were significant compared to the control. In fact, 25%-MIC HA showed the lowest mean fold-change value at 0.18 ± 0.08 among all materials *p* = 0.003 (Table S2 and Figure 5).

#### 3.2.3. hagA

Overall, all three materials have significantly reduced hagA expression by 0.30 ± 0.18, 0.66 ± 0.50, and 0.44 ± 0.25 folds for AZM, CHX, and HA, consecutively. The only significant difference between the three materials was observed between AZM and CHX *p* = 0.014 (Table S1 and Figure 3).

**Figure 6:**
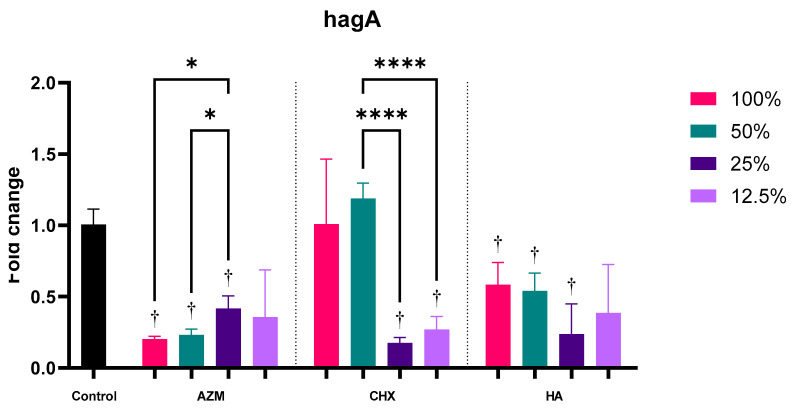
Fold-change values (mean ± SD) of different concentrations on expression of hagA. >0.05 (ns), ≤0.05 (*), ≤0.01 (**), ≤0.001 (***), ≤0.0001 (****). Statistically significant compared to control *p* < 0.05 (†).

Both AZM and HA have significantly reduced the expression of hagA at their three higher concentrations, while 12.5% MIC was not significant compared to the control. The higher two concentrations of CHX showed largely no change in hagA expression (1.01 ± 0.46 and 1.19 ± 0.11), while the lower two concentrations have reduced its expression significantly *p* = 0.000 (Table S2 and Figure 6).

#### 3.2.4. rgpA

Both AZM and HA have demonstrated a significant reduction in rgpA expression by 0.47 ± 0.38 and 0.67 ± 0.46 folds regardless of concentration. CHX, on the other hand, has shown a slight—but not significant—increase in its expression by 1.20 ± 1.09 folds. The difference between AZM and CHX was statistically significant *p* = 0.022. No significant difference was observed between AZM and HA nor CHX and HA (Table S1 and Figure 3).

**Figure 7:**
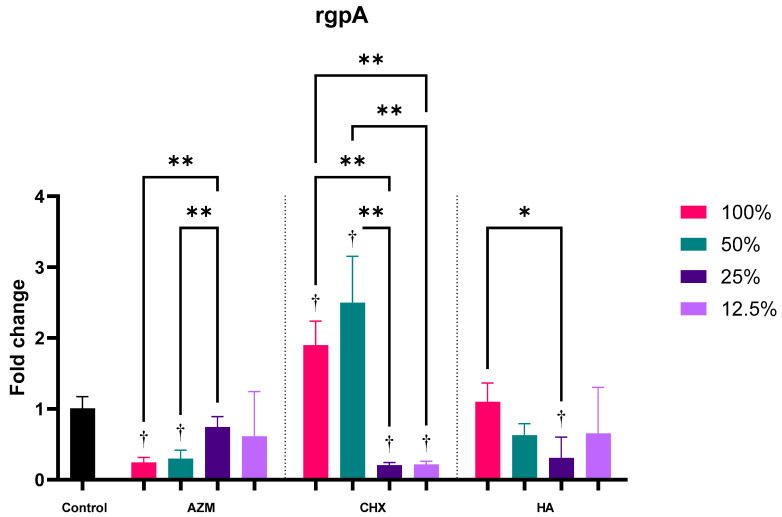
Fold-change values (mean ± SD) of different concentrations on expression of rgpA. >0.05 (ns), ≤0.05 (*), ≤0.01 (**), ≤0.001 (***), ≤0.0001 (****). Statistically significant compared to control *p* < 0.05 (†).

All AZM concentrations have reduced the expression of rgpA, but only 100% MIC and 50% MIC were significant compared to the control *p* = 0.000. Higher and lower concentrations of CHX demonstrated a contrasting behavior. Higher concentrations of CHX have significantly increased the induction of rgpA (1.90 ± 0.34 and 2.50 ± 0.65), while lower concentrations have significantly reduced its expression (0.20 ± 0.04 and 0.22 ± 0.04). MIC concentration of HA did not show changes in rgpA regulation, while all sub-MIC concentrations have reduced its expression. Only 25%-MIC HA was significant compared to the control *p* = 0.025 (Table S2 and Figure 7).

#### 3.2.5. rgpB

AZM and HA have reduced rgpB expression by 0.37 ± 0.25 and 0.48 ± 0.33 folds, but only the reduction by AZM was significant compared to the control *p* = 0.033. CHX on the other hand demonstrated almost no change in the level of expression compared to the control at 1.05 ± 1.02 folds. The difference between AZM and CHX was significant *p* = 0.018. The difference between AZM and HA was not statistically significant *p* = 0.527 (Table S1 and Figure 3).

**Figure 8:**
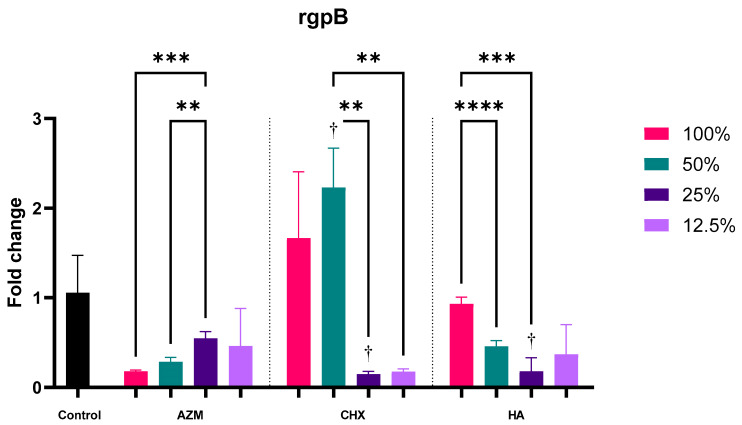
Fold-change values (mean ± SD) of different concentrations on expression of rgpB. >0.05 (ns), ≤0.05 (*), ≤0.01 (**), ≤0.001 (***), ≤0.0001 (****). Statistically significant compared to control *p* < 0.05 (†).

All concentrations of AZM decreased the expression of rgpB but none were significant compared to the control. Higher concentrations of CHX showed again an increase in rgpB induction while lower concentrations decreased its induction. Moreover, 50%-MIC CHX increased the induction of rgpB significantly by 2.23 ± 0.44 folds, while 25% significantly decreased its induction by 0.15 ± 0.03 folds. MIC concentration of HA did not show changes in rgpB regulation (0.93 ± 0.08), while all sub-MIC concentrations reduced its expression by 0.46 ± 0.06, 0.18 ± 0.15 and 0.37 ± 0.33 folds, consecutively. Only 25%-MIC HA was significant compared to the control *p* = 0.049 (Table S2 and Figure 8).

#### 3.2.6. kgp

AZM, CHX and HA reduced kgp expression by 0.21 ± 0.13, 0.76 ± 0.47 and 0.35 ± 0.22 folds, respectively. The reductions by AZM and HA were statistically significant compared to the control (*p* = 0.007 and 0.013). CHX’s impact on kgp induction was significantly less than AZM’s and HA’s. Furthermore, there was no significant difference between AZM and HA *p* = 0.055 (Table S1 and Figure 3).

**Figure 9:**
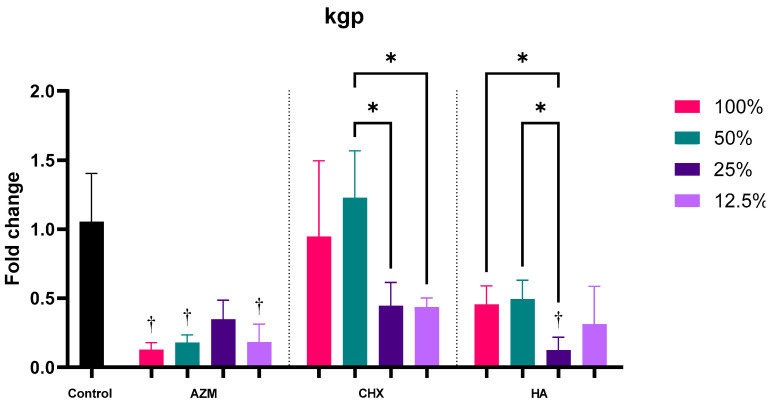
Fold-change values (mean ± SD) of different concentrations on expression of kgp. >0.05 (ns), ≤0.05 (*), ≤0.01 (**), ≤0.001 (***), ≤0.0001 (****). Statistically significant compared to control *p* < 0.05 (†).

All concentrations of AZM reduced kgp expression significantly except for 25% MIC which was not significant *p* = 0.058. Only the two lower concentrations of CHX were able to reduce kgp expression but none of the concentrations were significantly different from the control. All concentrations of HA reduced kgp expression by 0.46 ± 0.13, 0.49 ± 0.14, 0.12 ± 0.09, and 0.31 ± 0.27 folds, consecutively, but only 25% MIC was significant compared to the control *p* = 0.017 (Table S2 and Figure 9).

## 4. Discussion

*P. gingivalis* plays an important role in the pathogenesis of periodontal/peri-implant diseases as it leads to dysbiosis of a complex multispecies biofilm [6]. It has also shown host modulation ability that gives itself and other constituents of the biofilm an advantage to flourish and leave the host more susceptible to infection [7]. With the rising concerns of antibiotic resistance, it is imperative that we look for new approaches to suppress pathogens and reduce our reliance on conventional antibiotics.

In the present study, we aimed to evaluate the effect of hyaluronic acid, azithromycin, and chlorhexidine on the expression of fimA, mfa1, hagA, rgpA, rgpB, and kgp which are related to the virulence and adhesion of *P. gingivalis*. The results have demonstrated that HA, a naturally occurring constituent of the body, could have a potent effect on the function of *P. gingivalis* by downregulating these genes. HA was able to reduce the expression of all genes combined by over a half fold 0.46 ± 0.33. Although this reduction was significantly less than AZM at 0.31 ± 0.24 folds (*p* = 0.000), it was significantly better than the one achieved by CHX at 0.84 ± 0.77 folds (*p* = 0.000). Other studies have also demonstrated an antimicrobial effect of HA comparable to that of CHX. Binshabaib and her colleagues have shown that 0.8% HA was able to reduce colony-forming units of *P. gingivalis* grown on glass slides more significantly than 0.2% CHX after 48 and 72 h [35]. Although they were measuring the growth of *P. gingivalis* and not gene expression, their results were similar to ours in that HA was more effective than CHX. Rodrigues and her colleague reached different results when they used a mouthwash containing 0.025% sodium hyaluronate and xylitol. This mouthwash did not show an effect on the number of colonies of *P. gingivalis*, while it suppressed the growth of *Aggregatibacter actinomycetemcomitans* and *Prevotella intermedia*. A mouthwash containing 0.2% CHX, on the other hand, was able to suppress all three species [37]. It is worth considering that Rodrigues et al. have conducted their experiment on clinical isolates and not the reference strain as in our study. They have also used a mouthwash that has a low concentration of HA. In a study evaluating treatments of peri-implant mucositis, patients who used an adjunctive mouthwash containing 0.2% chlorhexidine gluconate with hyaluronic acid were associated with less detection frequency of *P. gingivalis* (26.7 ± 11.4) after one month of treatment compared to those who used a mouthwash containing 0.05% chlorhexidine gluconate only (40.00 ± 12.6) and those who did not use adjunctive mouthwash at all (43.8 ± 12.4) [38]. In another study, mouthwashes containing 0.2% chlorhexidine gluconate were able to reduce plaque formation after 4 days without brushing with a mean plaque index of 1.64 ± 0.31. This inhibition was slightly better than the one achieved by mouthwashes containing 0.025% of HA and 7.5% xylitol with a mean plaque index of 1.81 ± 0.21 [39]. It is difficult to interpret the role of HA from the previous studies since they have used commercial products where HA is available in variable concentrations and mostly mixed with other ingredients that could superimpose its effect.

In the present study, HA significantly downregulated all the investigated genes except rgpB, despite achieving a mean of a half fold 0.48 ± 0.33 (*p* = 0.068). It is interesting that the difference between HA and AZM was not significant for all the genes except fimA (*p* = 0.026). It was not anticipated that HA would exhibit such a potent effect on gene expression, similar to that of an antibiotic, albeit lacking the bactericidal strength of an antibiotic. Nevertheless, AZM demonstrated the lowest mean fold-change values with every gene. Kan et al. showed that sub-MIC AZM significantly decreased the expression of fimA, hagA, rgpA, and kgp but not mfa1 and rgpB [40]. Contrary to their findings, our study shows that AZM was able to significantly downregulate all these genes including mfa1 and rgpB. It is worth considering that the MIC in our study is higher than the one in their study (1.6 vs. 0.4 µg/mL). This could be attributed to the variation in culture media, growth conditions, and our decision to avoid using commonly used solvents—such as dimethyl sulfoxide—that inherently have antimicrobial properties [41].

The most effective concentration of HA in this study was 25% MIC or 1 mg/mL. It was the only concentration that significantly reduced the expression of all genes at the same time. Pirnazar and his colleagues suggested that HA exhibited a bacteriostatic effect on *P. gingivalis* at a high molecular weight and high concentrations [34]. It is surprising in our study that lower concentrations of HA showed greater downregulation almost every time. This behavior was more profound with CHX and nearly opposite to what was demonstrated by AZM. The higher two concentrations of CHX, in fact, upregulated the expression of rgpA and rgpB significantly. It can be speculated that this observation is related to survival or possibly antimicrobial resistance as gingipains are mainly responsible for the proteolytic activity [42].

Our study is the first to evaluate the effect of high molecular weight HA on the expression levels of genes related to adhesion and virulence in *P. gingivalis*. It is also the first to directly compare HA to an antibiotic. Our study derives its strength from its novelty and the use of pure research-grade sodium hyaluronate, unlike most available studies where HA is mixed with other compounds. However, this study has several limitations. The nature of in vitro studies makes it difficult to interpret the results clinically. Additionally, the response of the reference strain of *P. gingivalis* to HA might not be completely imitated by clinical strains. Finally, the protective nature of a microbial biofilm could diminish the effect of antimicrobials on its constituents.

## 5. Conclusions

High molecular weight hyaluronic acid (HA) has a potent effect on *P. gingivalis* by downregulating fimA, mfa1, hagA, rgpA, and kgp which are genes related to its function and attachment. The effect of hyaluronic acid is less than that of azithromycin and superior to chlorhexidine. Lower concentrations of hyaluronic acid were associated with an increased gene suppression, while the most effective concentration of HA was 1 mg/mL.

## Figures and Tables

**Table 1:** The sequence of primers used in this study.

Gene	Sequence	
16S rRNA	F	AGTCGCGTGAAGGAAGACTG
	R	TACCGAACAACCTACGCACC
fimA	F	TGTTGGGACTTGCTGCTCTT
	R	TTCGTCATCGCCAACTCCAA
mfa1	F	GATCCTGCAACCCACAATGC
	R	AGCCTGAGCCTGAGTAGACA
hagA	F	CCGCGAGATTCTGGGCAATA
	R	CCTGCTCCGATGAACTTGGT
rgpA	F	GTTCCATCACCGCTACCCAT
	R	GGACAAGGACCGACGAAAGA
rgpB	F	CGTCTTGCCTTCAGTAGCGA
	R	TGTAGAAAGTCCTGCTGCCG
kgp	F	GACCCTGCGTTGTAGCAGT
	R	GGTGTTGCTAATGCCAGCG

**Table 2:** Overall fold-change values for all genes combined.

	Mean	Std. Deviation	ANOVA (*p*-Value)	95% Confidence Interval for Mean		Games-Howell Post-Hoc Test (*p*-Value)		
				Lower Bound	Upper Bound	CHX	HA	Control
AZM	0.31	0.24	0.000	0.2686	0.3462	0.000	0.000	0.000
CHX	0.84	0.77		0.7134	0.9679		0.000	0.074
HA	0.46	0.33		0.4066	0.5151			0.000
Control	1.03	0.24		0.9445	1.106			

## Data Availability

All datasets generated during the current study were made available to the journal and will be provided without restrictions upon communications with the corresponding author.

## Supplementary Materials

The following supporting information can be downloaded at: https://www.mdpi.com/article/10.3390/pharmaceutics14081628/s1, Table S1: Overall fold-change values for independent genes, Table S2: Concentration fold-change values for independent genes.
