# Supplementary material for: High Molecular Weight Hyaluronic Acid Reduces the Expression of Virulence Genes fimA, mfa1, hagA, rgpA, and kgp in the Oral Pathogen Porphyromonas gingivalis
Source: Pharmaceutics. 2022 Aug 4;14(8):1628. doi: 10.3390/pharmaceutics14081628 (PMC9415305; doi:10.3390/pharmaceutics14081628)
Supplement: Supplementary file 1 [file pharmaceutics-14-01628-s001.zip › pharmaceutics-1819972-supplementary.pdf]

Table S1. Overall fold-change values for independent genes.

		**Mean**	**Std. Deviation**	ANOVA (P-value)	95% Confidence Interval for Mean		Games-Howell post-hoc test (P-value)			
					Lower Bound	Upper Bound	AZM	CHX	HA	Control
**FimA**	**Azm**	**0.20**	**0.15**	0.000	0.1428	0.2656		0.000	0.026	0.000
	CHX	0.49	0.23		0.3959	0.591	0.000		0.131	0.000
	HA	0.35	0.20		0.2687	0.4389	0.026	0.131		0.000
	Control	1.00	0.05		0.9459	1.0564	0.000	0.000	0.000	
Mfa1	Azm	0.28	0.13	0.000	0.2307	0.3388		0.007	0.092	0.002
	CHX	0.87	0.78		0.5387	1.1937	0.007		0.125	0.825
	HA	0.47	0.35		0.3224	0.6192	0.092	0.125		0.003
	Control	1.03	0.24		0.7763	1.276	0.002	0.825	0.003	
HagA	Azm	0.30	0.18	0.000	0.2238	0.38		0.014	0.163	0.000
	CHX	0.66	0.50		0.4476	0.874	0.014		0.228	0.023
	HA	0.44	0.25		0.3309	0.5426	0.163	0.228		0.000
	Control	1.01	0.11		0.891	1.1191	0.000	0.023	0.000	
RgpA	Azm	0.47	0.38	0.004	0.3162	0.6335		0.022	0.380	0.000
	CHX	1.20	1.09		0.7426	1.6661	0.022		0.147	0.838
	HA	0.67	0.46		0.476	0.8681	0.380	0.147		0.035
	Control	1.01	0.16		0.8387	1.1815	0.000	0.838	0.035	
RgpB	Azm	0.37	0.25	0.001	0.264	0.4737		0.018	0.527	0.033
	CHX	1.05	1.02		0.625	1.4849	0.018		0.065	1.000
	HA	0.48	0.33		0.3441	0.6253	0.527	0.065		0.068
	Control	1.06	0.42		0.6171	1.4939	0.033	1.000	0.068	
Kgp	Azm	0.21	0.13	0.000	0.1561	0.2635		0.000	0.055	0.007
	CHX	0.76	0.47		0.5676	0.9607	0.000		0.002	0.379
	HA	0.35	0.22		0.2544	0.4394	0.055	0.002		0.013
	Control	1.05	0.35		0.6864	1.4209	0.007	0.379	0.013	

Table S2. Concentration fold-change values for independent genes.

		**Mean**	Std. Deviation	ANOVA (P-value)	95% Confidence Interval for Mean		Games-Howell post-hoc test (P-value)				
					Lower Bound	Upper Bound	100%	50%	25%	12.5%	Control
					**FimA**						
**AZM**	**100%**	**0.07**	0.01	0.000	0.0668	0.0784		0.000	0.001	0.812	0.000
	50%	0.16	0.02		0.1465	0.1815	0.000		0.002	1.000	0.000
	25%	0.36	0.05		0.3052	0.4096	0.001	0.002		0.897	0.000
	12.5%	0.22	0.21		0.0044	0.4411	0.812	1.000	0.897		0.003
CHX	100%	0.62	0.29	0.000	0.3168	0.9276		1.000	0.377	0.754	0.285
	50%	0.69	0.07		0.6149	0.7712	1.000		0.000	0.007	0.001
	25%	0.28	0.08		0.1993	0.3568	0.377	0.000		0.746	0.000
	12.5%	0.38	0.11		0.2699	0.4911	0.754	0.007	0.746		0.000
HA	100%	0.37	0.05	0.000	0.3157	0.4214		0.191	0.830	1.000	0.000
	50%	0.49	0.07		0.4106	0.5666	0.191		0.299	0.979	0.000
	25%	0.22	0.21		0.0022	0.4351	0.830	0.299		0.999	0.003
	12.5%	0.34	0.30		0.0213	0.6579	1.000	0.979	0.999		0.045
					Mfa1						
AZM	100%	0.25	0.12	0.000	0.1256	0.3735		1.000	0.745	1.000	0.004
	50%	0.21	0.07		0.1374	0.2814	1.000		0.374	0.848	0.010
	25%	0.41	0.16		0.238	0.5808	0.745	0.374		0.725	0.016
	12.5%	0.27	0.05		0.2141	0.3273	1.000	0.848	0.725		0.008
CHX	100%	1.15	0.31		0.8286	1.4808		0.990	0.022	0.011	0.999
	50%	1.63	1.07		0.5133	2.7504	0.990		0.386	0.307	0.946
	25%	0.39	0.21		0.1728	0.6134	0.022	0.386		0.989	0.020
	12.5%	0.29	0.12		0.163	0.4075	0.011	0.307	0.989		0.005
HA	100%	0.83	0.39		0.4244	1.2336		0.971	0.123	0.272	0.992
	50%	0.56	0.34		0.1956	0.9181	0.971		0.440	0.849	0.355
	25%	0.18	0.08		0.0967	0.2577	0.123	0.440		0.087	0.003
	12.5%	0.32	0.04		0.2765	0.3637	0.272	0.849	0.087		0.012
					HagA						
AZM	100%	0.20	0.02	0.000	0.18	0.224		0.859	0.025	0.981	0.000
	50%	0.23	0.04		0.1913	0.2738	0.859		0.045	0.996	0.000

	25%	0.42	0.09		0.3261	0.5088	0.025	0.045		1.000	0.000
	12.5%	0.36	0.33		0.0067	0.7047	0.981	0.996	1.000		0.068
CHX	100%	1.01	0.46		0.5304	1.4865		0.995	0.094	0.143	1.000
	50%	1.19	0.11		1.0746	1.3019	0.995		0.000	0.000	0.279
	25%	0.18	0.04		0.1365	0.2155	0.094	0.000		0.537	0.000
	12.5%	0.27	0.09		0.176	0.3651	0.143	0.000	0.537		0.000
HA	100%	0.58	0.15		0.4216	0.7467		1.000	0.195	0.963	0.012
	50%	0.54	0.13		0.408	0.6719	1.000		0.270	0.991	0.002
	25%	0.24	0.21		0.0154	0.4593	0.195	0.270		0.998	0.002
	12.5%	0.39	0.34		0.0299	0.7414	0.963	0.991	0.998		0.090
RgpA											
AZM	100%	0.24	0.07		0.1706	0.3176		0.996	0.003	0.927	0.000
	50%	0.30	0.12		0.1752	0.4208	0.996		0.007	0.972	0.000
	25%	0.74	0.15		0.5872	0.8984	0.003	0.007		1.000	0.265
	12.5%	0.61	0.63		-0.0474	1.2765	0.927	0.972	1.000		0.914
CHX	100%	1.90	0.34		1.5438	2.254		0.718	0.001	0.001	0.014
	50%	2.50	0.65	0.000	1.8118	3.1833	0.718		0.006	0.006	0.036
	25%	0.20	0.04		0.1645	0.2448	0.001	0.006		1.000	0.001
	12.5%	0.22	0.04		0.1706	0.2621	0.001	0.006	1.000		0.001
HA	100%	1.10	0.27		0.8205	1.3779		0.117	0.020	0.900	1.000
	50%	0.63	0.16		0.4552	0.7992	0.117		0.559	1.000	0.061
	25%	0.31	0.29		-0.0005	0.6181	0.020	0.559		0.981	0.025
	12.5%	0.65	0.65		-0.0327	1.3388	0.900	1.000	0.981		0.959
RgpB											
AZM	100%	0.18	0.01		0.1642	0.1945		0.055	0.001	0.852	0.056
	50%	0.29	0.05		0.2312	0.3391	0.055		0.002	0.990	0.090
	25%	0.55	0.08		0.4682	0.6275	0.001	0.002		1.000	0.344
	12.5%	0.46	0.42		0.0249	0.9012	0.852	0.990	1.000		0.477
CHX	100%	1.67	0.74	0.000	0.8891	2.443		0.886	0.061	0.065	0.827
	50%	2.23	0.44		1.7711	2.6916	0.886		0.001	0.002	0.023
	25%	0.15	0.03		0.1148	0.1804	0.061	0.001		0.932	0.048
	12.5%	0.17	0.03		0.1408	0.2086	0.065	0.002	0.932		0.054
HA	100%	0.93	0.08		0.8519	1.0094		0.000	0.000	0.122	0.999

	50%	0.46	0.06		0.3923	0.5258	0.000		0.088	1.000	0.216
	25%	0.18	0.15		0.0198	0.3392	0.000	0.088		0.968	0.049
	12.5%	0.37	0.33		0.0212	0.7179	0.122	1.000	0.968		0.214
Kgp											
AZM	100%	0.13	0.05		0.0758	0.1809		0.870	0.152	0.995	0.020
	50%	0.18	0.06		0.1208	0.2381	0.870		0.367	1.000	0.026
	25%	0.35	0.14		0.2041	0.4927	0.152	0.367		0.643	0.058
	12.5%	0.18	0.13		0.0464	0.3197	0.995	1.000	0.643		0.021
CHX	100%	0.95	0.55		0.3687	1.5232		0.992	0.656	0.602	1.000
	50%	1.23	0.34	0.000	0.8693	1.5841	0.992		0.031	0.035	0.999
	25%	0.45	0.17		0.2706	0.6236	0.656	0.031		1.000	0.116
	12.5%	0.44	0.07		0.3679	0.5056	0.602	0.035	1.000		0.105
HA	100%	0.46	0.13		0.315	0.5959		1.000	0.022	0.984	0.118
	50%	0.49	0.14		0.3523	0.6371	1.000		0.012	0.929	0.154
	25%	0.12	0.09		0.0256	0.2232	0.022	0.012		0.881	0.017
	12.5%	0.31	0.27		0.0264	0.5996	0.984	0.929	0.881		0.063
